# Deglacial Subantarctic CO_2_ outgassing driven by a weakened solubility pump

**DOI:** 10.1038/s41467-022-32895-9

**Published:** 2022-09-03

**Authors:** Yuhao Dai, Jimin Yu, Haojia Ren, Xuan Ji

**Affiliations:** 1 Research School of Earth Sciences, The Australian National University, Canberra, ACT Australia; 2 Pilot National Laboratory for Marine Science and Technology (Qingdao), Qingdao, China; 3 Department of Geosciences, National Taiwan University, Taipei, Taiwan; 4 Present Address: Department of Geology, Lund University, Lund, Sweden

**Keywords:** Palaeoceanography, Carbon cycle

## Abstract

The Subantarctic Southern Ocean has long been thought to be an important contributor to increases in atmospheric carbon dioxide partial pressure (pCO_2_) during glacial-interglacial transitions. Extensive studies suggest that a weakened biological pump, a process associated with nutrient utilization efficiency, drove up surface-water pCO_2_ in this region during deglaciations. By contrast, regional influences of the solubility pump, a process mainly linked to temperature variations, have been largely overlooked. Here, we evaluate relative roles of the biological and solubility pumps in determining surface-water pCO_2_ variabilities in the Subantarctic Southern Ocean during the last deglaciation, based on paired reconstructions of surface-water pCO_2_, temperature, and nutrient utilization efficiency. We show that compared to the biological pump, the solubility pump imposed a strong impact on deglacial Subantarctic surface-water pCO_2_ variabilities. Our findings therefore reveal a previously underappreciated role of the solubility pump in modulating deglacial Subantarctic CO_2_ release and possibly past atmospheric pCO_2_ fluctuations.

## Abstract (web summary)

Using paired reconstructions of seawater pCO_2_, temperature, and nutrient utilization, Dai et al. show underappreciated influences of the solubility pump on deglacial Subantarctic surface-water pCO_2_ variabilities compared to the biological pump.

## Introduction

The Southern Ocean is widely regarded as a crucial source of carbon dioxide (CO_2_) to the atmosphere during glacial terminations because this region serves as a window for CO_2_ exchanges between the atmosphere and the ocean interior^1–4^. In the Southern Ocean, prevailing southern hemisphere westerly winds drive upwelling of carbon-rich deep waters surrounding Antarctica, some of which are transported northward to the Subantarctic Zone (SAZ)^5,6^. The surface SAZ exposes the newly upwelled carbon-rich deep waters to the atmosphere enabling CO_2_ outgassing, before these waters are entrained to form intermediate and mode waters^5–7^.

Changes in the SAZ have been thought to be critical to deglacial atmospheric pCO_2_ rises, with a contribution estimated to be around 40 ppm^8–11^. In the SAZ, CO_2_ tends to escape to the atmosphere due to elevated surface-water CO_2_ partial pressures (pCO_2_) driven by high surface-water dissolved inorganic carbon (DIC) concentrations^12,13^ associated with the newly upwelled deep waters surrounding Antarctica. CO_2_ outgassing in the SAZ is somewhat alleviated by biologically driven carbon sequestration that exports carbon to depths in the form of organic matter, a process called the biological pump^1,14,15^. In addition to this biological process, it is important to note that surface-water pCO_2_ is further affected by CO_2_ solubility determined by seawater temperature and salinity, a process known as the solubility pump^14,16^. Changes in the solubility pump have been shown by modeling studies to contribute substantially to atmospheric pCO_2_ variability^2,17–19^. Everything else being equal, warming lowers CO_2_ solubility in seawater and thus increases surface-water pCO_2_ with an effect to cause CO_2_ outgassing from the ocean to the atmosphere^16^. Surface-water pCO_2_ increases with increasing salinity, but the salinity effect on the CO_2_ solubility is generally smaller than the temperature effect in most regions^20^ (Supplementary Fig. 2).

During glacial terminations, it has been proposed that the biological pump efficiency was lowered, driving up CO_2_ outgassing in the SAZ^1,11,21–23^. The deglacial SAZ biological pump efficiency decline has been linked to reduced supplies of micronutrients such as iron via eolian lithogenic fluxes^10,11,24^. In this case, weakened biological pump would leave more carbon unused in the SAZ surface, raising surface-water pCO_2_ which would stimulate CO_2_ outgassing^1^. On the other hand, as manifested by Southern Ocean temperature reconstructions^25–27^, deglacial SAZ warming, in theory, should weaken the solubility pump, raise surface-water pCO_2_, and promote CO_2_ outgassing^16^ (Supplementary Fig. 1). Existing reconstructions in the SAZ indeed show elevated surface-water pCO_2_ during the last deglaciation (~18-11 ka BP)^22,23^. Yet, it remains unknown regarding the respective roles of biological and solubility pump changes in affecting these past surface-water pCO_2_ rises in the SAZ and by extension atmospheric pCO_2_ changes.

Here, we systematically investigate the contributions of the biological and solubility pumps to the SAZ surface-water pCO_2_ changes in the modern ocean and during the last deglaciation. For the deglacial investigation, we have generated a surface-water pCO_2_ record using a sediment core from the Southwest Pacific, paired with nutrient utilization efficiency and sea surface temperature (SST) reconstructions. We evaluate the relative roles of biological and solubility pumps in regulating deglacial surface-water pCO_2_ changes at our site location. The same approach is further applied to published records at three additional SAZ locations. Moreover, we examine simulated early deglacial carbon cycle changes in an earth system model^28^ to distinguish effects of the two pumps during the early deglaciation. All our investigations suggest a strong solubility pump effect on the SAZ surface-water pCO_2_ changes, urging a rethinking of mechanisms underlying deglacial CO_2_ outgassing from the SAZ and, by extension, past atmospheric pCO_2_ variabilities.

## Results

### Solubility pump in the modern SAZ

**Fig. 1:**
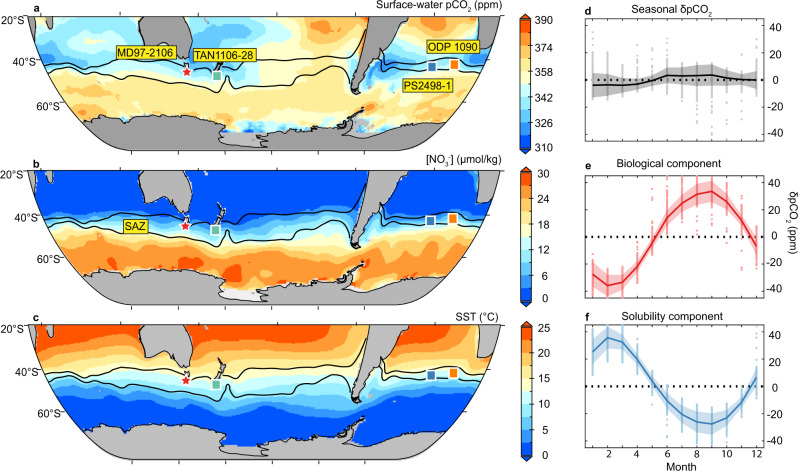
Surface-water chemistry in the modern Southern Ocean. **a** Annual mean surface-water pCO_2_ during years 1985–2018. The map is made from data presented in Gregor and Gruber^29^. https://creativecommons.org/licenses/by/4.0/. **b** Annual mean surface-water nitrate concentration. The map is made from data presented in Garcia et al.^48^, accessible from https://www.ncei.noaa.gov/access/world-ocean-atlas-2018/. **c** Annual mean sea surface temperature. Two black curves indicate the modern positions of the Subtropical Front (STF; the northern curve) and the Subantarctic Front (SAF; the southern curve), respectively, and the region between them is the Subantarctic Zone (SAZ). The red star represents the location of our study site MD97-2106, and squares represent locations with published δ^11^B-based surface-water pCO_2_ reconstructions in the SAZ. The map is made from data presented in Locarnini et al.^49^, accessible from https://www.ncei.noaa.gov/access/world-ocean-atlas-2018/. **d** Monthly surface-water pCO_2_ variability within the SAZ (year 1985–2018) calculated from the OceanSODA-ETHZ dataset^29^. **e** Monthly surface-water pCO_2_ variability attributed to biological pump changes within the SAZ calculated from the OceanSODA-ETHZ dataset^29^. **f** Monthly surface-water pCO_2_ variability attributed to solubility pump changes within the SAZ calculated from the OceanSODA-ETHZ dataset^29^. In **d–f**, shadings show ±1σ standard deviation ranges of observations at discrete locations (represented by dots). As can be seen from **d–f**, the solubility pump plays a critical role in stabilizing SAZ surface-water pCO_2_ by countering effects due to biological pump changes.

In the modern SAZ, both biological and solubility pumps strongly control spatial distribution and seasonal variability of surface-water pCO_2_^12,13,29^. Regarding the spatial distribution, Fig. 1a shows small northward declines in annual mean surface-water pCO_2_ within the SAZ, where nutrient (nitrate) is progressively utilized equatorward (Fig. 1b). Enhanced nutrient consumption would lower surface-water pCO_2_, but this biological effect is compensated by the opposing influence of equatorward surface-water warming in the SAZ (Fig. 1c). Consequently, surface-water pCO_2_ changes caused by nutrient consumption is largely balanced by decreasing CO_2_ solubility, and the total northward surface-water pCO_2_ decline is marginal. Regarding the seasonal variability, Fig. 1d shows minimal monthly surface-water pCO_2_ deviation from the annual mean levels in the SAZ. By contrast, seasonal changes in the biological and solubility pumps each cause surface-water pCO_2_ to fluctuate ~40 ppm around the annual mean level (Fig. 1e, f)^12,29^. Because the strong (weak) biological pump occurs during warm (cold) seasons with low (high) CO_2_ solubility, effects from the biological and solubility pumps generally cancel each other, leading to little seasonal surface-water pCO_2_ variability. Combined, these spatial and temporal patterns suggest that the solubility pump plays a critical role, comparable to that of the biological pump, in determining the SAZ surface-water pCO_2_ fluctuations in the modern ocean. Next, we move on to explore the impact of the solubility pump on past surface-water pCO_2_ changes, a topic that has rarely been studied by proxy reconstructions.

### New deglacial SAZ surface-water pCO_2_ and δ^15^N records

**Fig. 2:**
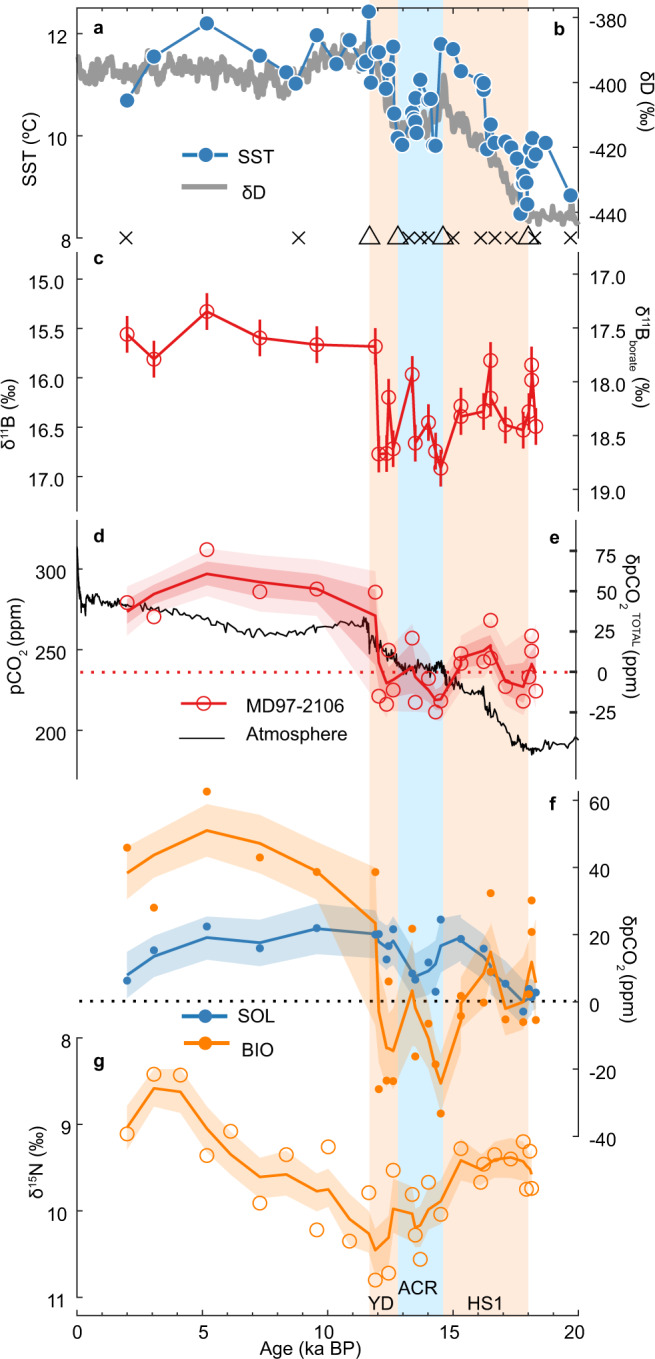
Surface-water reconstructions at site MD97-2106. **a** Sea surface temperature (SST) reconstructed from planktic foraminiferal Mg/Ca^36^ (blue circles, left axis). **b** Antarctic temperature changes represented by δD^26^ (gray curve, right axis). Crosses and triangles at the bottom are age control points based on planktic radiocarbon dating and SST-δD matching, respectively. **c** Planktic foraminiferal δ^11^B and seawater borate δ^11^B with error bars showing ±2σ uncertainties. **d** Reconstructed surface-water pCO_2_ at site MD97-2106 (red circles) compared with atmospheric pCO_2_ recorded in the Antarctic ice cores (black curve)^43, 44^ (left axis). **e** Total surface-water pCO_2_ change relative to 18 ka (δpCO_2_^TOTAL^) at site MD97-2106 (right axis). **f** Deglacial surface-water pCO_2_ attributed to the solubility and biological pumps (δpCO_2_^SOL^ and δpCO_2_^BIO^, blue and orange dots, respectively) at site MD97-2106. **g** Foraminifera-bound δ^15^N, a proxy reflecting surface nutrient utilization. Curves in **d**–**g** are derived from a LOESS smoother. In **d**, light and dark envelopes, respectively, represent 2.3–97.7% (roughly ±2σ) and 15.9–84.1% (roughly ±1σ) uncertainty ranges of timeseries incorporating uncertainties from measurements, all individual parameters used for calculations, and age models. In **f** and **g** only 15.9–84.1% uncertainty ranges are shown for clarify. The vertical pale orange bars represent Heinrich Stadial 1 (HS1; ~18.0–14.6 ka BP) and the Younger Dryas (YD; ~12.8–11.7 ka BP). The vertical pale blue bar represents the Antarctic Cold Reversal (ACR; ~14.6–12.8 ka BP). δpCO_2_^SOL^ and δpCO_2_^BIO^ (**f**) show similar structures to SST (**a**) and foraminifera-bound δ^15^N (**g**), respectively.

We present records of surface-water pCO_2_ and nutrient utilization, respectively, based on boron isotopes (δ^11^B)^22,30,31^ and foraminifera-bound nitrogen isotopes (δ^15^N_FB_) of mixed-layer-dwelling planktic foraminifera species *Globigerina bulloides* for site MD97-2106 from the Southwest Pacific Ocean (Fig. 1). Site MD97-2106 (45.15°S, 146.28°E) is located in the northern part of the SAZ. Compared to today, the Subtropical Front, the northern boundary of the SAZ, possibly shifted northward to Tasmania during the Last Glacial Maximum (LGM; ~22-18 ka)^32,33^. During the last deglaciation, the Subtropical Front might migrate southwards^32,33^, but unlikely to the south of our site. This is because the Subtropical Front marks a ~4 °C SST gradient^34^, while reconstructions at our site only show ~3 °C SST change during the entire deglaciation^35,36^. Consequently, our site was likely located within the SAZ during the entire last deglaciation, ideal for investigating deglacial SAZ surface conditions. The age model of site MD97-2106 during the last deglaciation is based on new radiocarbon dates and tuning of SST at this site to Antarctic temperatures^26^ (Fig. 2a, b; Supplementary Table 1, Supplementary Fig. 4). See Methods for analytical details.

Our reconstructed surface-water pCO_2_ at site MD97-2106 fluctuated between ~210 and ~270 ppm during the last deglaciation, reached ~270 ppm at the onset of the Holocene, and increased by ~20 ppm from the early to middle Holocene (Fig. 2d). The range of deglacial surface-water pCO_2_ at site MD97-2106 is comparable to previous reconstructions^22,23^. At our site, surface-water pCO_2_ briefly dropped below the atmospheric pCO_2_ values during the Antarctic Cold Reversal (ACR; 14.6–12.8 ka BP) and the Younger Dryas (YD; 12.8–11.7 ka BP) (Fig. 2d).

In addition to our surface-water pCO_2_ reconstructions, we employ δ^15^N_FB_ to infer nitrate utilization efficiency at site MD97-2106. δ^15^N_FB_ reflects δ^15^N of surface-water nitrate taken up by foraminifera which increases as surface nitrate is progressively consumed by photosynthetic algae^11,37,38^. During Heinrich Stadial 1 (HS1; 18.0-14.6 ka BP) when the age model of our core is well constrained by a warming phase, δ^15^N_FB_ at our site remained roughly unchanged (Fig. 2b, g). This observation is consistent with a precisely dated coral-bound δ^15^N record from the same region showing minimal δ^15^N variability over the same period^21^ (Supplementary Fig. 9). Maxima of δ^15^N_FB_ occurred during the ACR and the YD, coinciding with surface-water pCO_2_ minima. During the Holocene, δ^15^N_FB_ declined by about 2.0‰, consistent with other fossil-bound δ^15^N records from the Southern Ocean^11,21,39–41^.

### Evaluating past biological and solubility effects at site MD97-2106

We partition surface-water pCO_2_ changes at site MD97-2106 into two components caused by changes in biological and solubility pumps, using a similar method previously applied to investigate modern surface-water pCO_2_ variability^12,16,29^. Firstly, we calculate total in-situ surface-water pCO_2_ changes (noted as δpCO_2_^TOTAL^) relative to the reference age of 18 ka; choosing a different reference age has little effect on our conclusions (Supplementary Fig. 6). Secondly, surface-water pCO_2_ is recalculated using carbonate chemistry (i.e., DIC and alkalinity) fixed at the reference age, but using varying SST and sea surface salinity (SSS) based on our reconstructions (Methods). Thirdly, this recalculated surface-water pCO_2_ is used to compute changes relative to 18 ka (the reference age). The relative surface-water pCO_2_ changes calculated in this way are only driven by SST and SSS, and thus are attributed to the solubility pump effect (noted as δpCO_2_^SOL^). Fourthly, we calculate the difference between δpCO_2_^TOTAL^ and δpCO_2_^SOL^, which is defined as the biology-driven surface-water pCO_2_ change (noted as δpCO_2_^BIO^). We also provide an alternative approach to directly calculate δpCO_2_^BIO^, which yields consistent results with those presented in the main text (Methods; Supplementary Fig. 8). Any influence of external processes on surface-water pCO_2_, such as changes associated with frontal shift, is embedded in our method, because these external processes affect surface-water pCO_2_ via the carbonate chemistry, SST-SSS, or both. See “Methods” for calculation details.

As can be seen from Fig. 2f, δpCO_2_^BIO^ at site MD97-2106 fluctuated between ~−20 ppm and ~+40 ppm from the LGM to the early Holocene, followed by a ~20-ppm increase during the Holocene. From 18 to 15 ka, δpCO_2_^BIO^ showed little net change, suggesting marginal influence of biological processes on surface-water pCO_2_ variations at our site. During the ACR and the YD, δpCO_2_^BIO^ exhibits mostly negative values, indicating a strengthened biological pump that would lower surface-water pCO_2_ during these times. The evolution of δpCO_2_^BIO^ at our site is well corroborated by our δ^15^N_FB_ record from the same core. Little net δpCO_2_^BIO^ change concurred with stable δ^15^N_FB_ during HS1, while negative δpCO_2_^BIO^ during the ACR and the YD coincided with higher δ^15^N_FB_ values which indicate more complete nutrient utilization (Fig. 2f–g). We note that, in addition to nitrate utilization in the SAZ, our δ^15^N_FB_ might have also been affected by δ^15^N of nitrate supplied to our site, which depends on the nitrate utilization of the nitrate source^11,21,41,42^. Despite these complications, similar deglacial structures in δ^15^N_FB_ and independently derived δpCO_2_^BIO^ suggest that δ^15^N_FB_ at site MD97-2106 reflects local nitrate utilization efficiency. Moreover, given their independent methods, consistent patterns of our δpCO_2_^BIO^ and δ^15^N_FB_ lend strong support to our inference about past biological pump changes at this site. In stark contrast to previous findings in the SAZ^22–24^, our δpCO_2_^BIO^ and δ^15^N_FB_ suggest that the biological pump played a minor role in contributing to surface-water pCO_2_ and thus air-sea CO_2_ exchange at site MD97-2106 during HS1 and the YD when atmospheric pCO_2_ rose substantially^43,44^.

Compared with δpCO_2_^BIO^, our calculated δpCO_2_^SOL^, which reflects solubility pump effects, shows a different history (Fig. 2f). As expected, deglacial δpCO_2_^SOL^ increased in response to warming, although this warming effect was slightly counteracted by a declining SSS (Supplementary Fig. 5). From 18 to 10 ka, δpCO_2_^SOL^ showed a net increase of ~20 ppm, contributing to about one-third of the δpCO_2_^TOTAL^ at site MD97-2106 over the same period. More specifically, the δpCO_2_^SOL^ increase dominated the δpCO_2_^TOTAL^ rise from 18 to 15 ka, in contrast to little net change in the concurrent δpCO_2_^BIO^. This highlights the critical role of the weakened solubility pump in maintaining positive sea-air pCO_2_ gradients, which would contribute to the atmospheric pCO_2_ rise during HS1. During the YD, rising δpCO_2_^SOL^ helped reverse biological effects (shown by δpCO_2_^BIO^ and δ^15^N_FB_) to limit the development of negative sea-air pCO_2_ gradient (Fig. 2f-g), contributing to the contemporary atmospheric pCO_2_ rise.

By separating influences of the biological and solubility pumps downcore, we demonstrate that a substantial portion of deglacial surface-water pCO_2_ rise at our site originated from variations in the solubility pump. This suggests that the solubility pump, which has been neglected in previous investigations in the region, played an important role in regulating deglacial surface-water pCO_2_ changes in the Pacific SAZ.

### Proxy and model data evaluation for broader SAZ

**Fig. 3:**
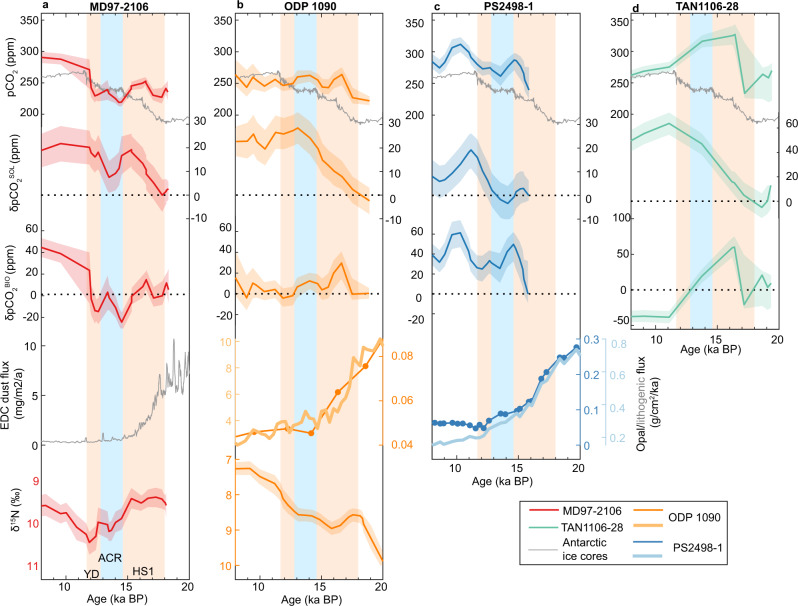
Deglacial surface-water pCO_2_, solubility and biological pump effects, dust and opal fluxes, and nutrient utilization at four Subantarctic Zone sites. **a** MD97-2106; **b** ODP 1090^11, 23, 46^; **c** PS2498-1^22, 47^; and **d** TAN1106-28^23^. Data in the four panels are arranged as follows. First row: surface-water pCO_2_ at investigated sites (red, orange, blue, and green curves) compared to atmospheric pCO_2_ recorded in Antarctic ice cores^43,44^ (gray curves). Second row: surface-water pCO_2_ change attributed to the solubility pumps (δpCO_2_^SOL^). Third row: surface-water pCO_2_ change attributed to the biological pump (δpCO_2_^BIO^). Fourth row: dust fluxes recorded in an Antarctic ice core^45^ (**a**), lithogenic (pale orange curve)^22^ and opal (orange curve with dots)^46^ fluxes at site ODP 1090 (**b**), lithogenic (pale blue curve) and opal (blue curve with dots) fluxes at site PS2498-1^47^ (**c**). Fifth row: Foraminifera-bound δ^15^N (this study and^11^). Note that scales of δpCO_2_^SOL^ and δpCO_2_^BIO^ differ in **d** compared to **a–c**. Envelopes represent 15.9–84.1% uncertainties incorporating uncertainties from measurements, all individual parameters required for calculations, and age models. The vertical pale orange bars represent Heinrich Stadial 1 (HS1; ~18.0–14.6 ka BP) and the Younger Dryas (YD; ~12.8–11.7 ka BP). The vertical pale blue bar represents the Antarctic Cold Reversal (ACR; ~14.6–12.8 ka BP). The reference age for relative surface-water pCO_2_ change decomposition at these sites is set at 18 ka, except at site PS2498-1, where it is set at the oldest age of ~15.8 ka. For all sites examined, solubility pump changes consistently contribute ~20–50 ppm to the deglacial surface-water pCO_2_ changes (second row). Surface-water pCO_2_ changes attributed to the biological pump (third row) differ from dust and export production (fourth row).

To quantify past influences of biological and solubility pumps in broader SAZ regions, we reanalyze deglacial surface-water pCO_2_ changes at three additional locations using published proxy records^22,23^ (Figs. 1a; 3b–d). At all the investigated sites, our calculated δpCO_2_^BIO^ suggests little-to-modest biological pump effects on SAZ surface-water pCO_2_ during times with large atmospheric pCO_2_ rises. For instance, except for a brief excursion at ~17 ka, δpCO_2_^BIO^ at Site ODP 1090 (Fig. 3b) varied little and suggests minimal biological pump effects on surface-water pCO_2_ during the last deglaciation^23^. At site PS2498-1, despite an overall larger δpCO_2_^BIO^ contribution to surface-water pCO_2_ compared to other sites, roughly stable average δpCO_2_^BIO^ during ~14-11 ka contributed little to the observed surface-water pCO_2_ rise (Fig. 3c). At site TAN1106-28, δpCO_2_^BIO^ showed a prominent peak at ~16 ka, but the general deglacial trend of δpCO_2_^BIO^ is poorly defined by the low temporal resolution. In contrast to δpCO_2_^BIO^ changes, our calculated δpCO_2_^SOL^ at all studied sites supports that solubility pump changes consistently contributed to deglacial δpCO_2_^TOTAL^. From 18 to 10 ka, we observe well-defined δpCO_2_^SOL^ increases of ~20 ppm at Site ODP 1090 from the South Atlantic. At site TAN1106-28 from the South Pacific, δpCO_2_^SOL^ increased by ~50 ppm, in response to the deglacial SST change of ~7 °C in part caused by a possible frontal shift over this site^23^. Although the record at site PS2498-1 does not cover the entire last deglaciation, δpCO_2_^SOL^ at this site shows a ~30-ppm increase during ~14–11 ka (Fig. 3c). These δpCO_2_^SOL^ changes significantly contribute to, or even dominate, the surface-water pCO_2_ variations at these sites, strengthening our findings at site MD97-2106. Overall, our analyses of proxy data from different sectors of the Southern Ocean demonstrate that deglacial surface-water pCO_2_ changes in the SAZ are substantially affected by solubility pump changes, rather than solely by biological pump changes as previously assumed^11,22,23^.

**Fig. 4:**
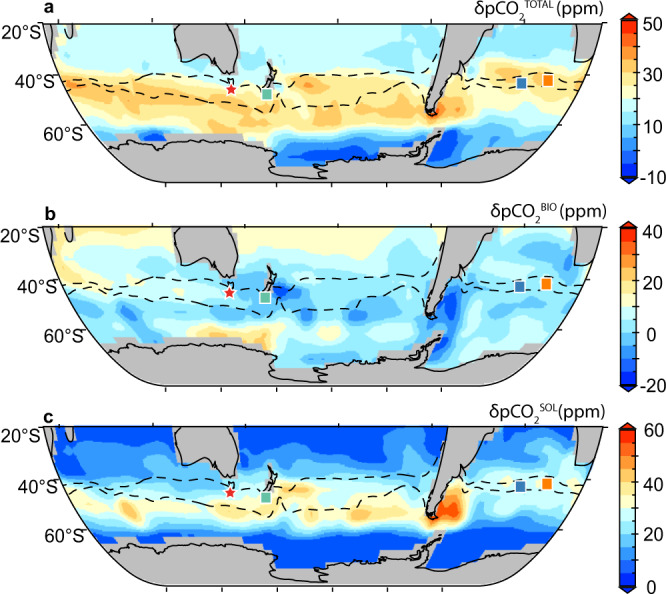
Southern Ocean surface-water pCO_2_ changes in a climate model simulation during the last deglaciation^28^. **a** Total surface-water pCO_2_ change (δpCO_2_^TOTAL^). **b** surface-water pCO_2_ change attributed to the biological pump (δpCO_2_^BIO^). **c**, surface-water pCO_2_ change attributed to the solubility pump (δpCO_2_^SOL^). All anomalies (δ) represent changes between 19 ka and 15 ka. Note different δpCO_2_ scales for the three panels. The region between the dotted lines is the modern Subantarctic Zone (SAZ). The locations of MD97-2106 (red star) and other investigated sites (ODP 1090, orange square; PS2498-1, blue square; TAN1106-28 teal square) are shown. As can be seen, the total δpCO_2_^TOTAL^ rise in the SAZ from 19 to 15 ka in this model simulation is mainly driven by solubility pump changes. The figure is made from recalculation based on data presented in Menviel et al.^28^.

We further scrutinize the role of the solubility pump in affecting deglacial SAZ surface-water pCO_2_ in a simulation by climate model LOVECLIM^28^. In this simulation, a 30-ppm atmospheric pCO_2_ increase is achieved during HS1 when the Southern Ocean overturning circulation and southern hemisphere westerly winds are intensified. The rising surface-water pCO_2_ in the Southern Ocean is diagnosed as a main CO_2_ source for the early deglacial atmospheric pCO_2_ increase^28^. Using the same method applied to proxy data, we quantify δpCO_2_^BIO^ and δpCO_2_^SOL^ changes between 19 ka and 15 ka in this simulation (“Methods”). Our decomposition (Fig. 4) reveals that δpCO_2_^BIO^ changes are either small or tend to lower pCO_2_^TOTAL^ in the SAZ. Because nutrient utilization forced by iron availability is not prescribed in the model, our calculated δpCO_2_^BIO^ per se cannot be used to dismiss iron fertilization effect on deglacial SAZ surface-water pCO_2_ changes. By contrast, δpCO_2_^SOL^ changed substantially due to strong surface warming, dominating surface-water δpCO_2_^TOTAL^ rise in the SAZ (Fig. 4). Therefore, our model data analyses suggest that solubility pump changes are crucial for deglacial surface-water pCO_2_ and air-sea CO_2_ exchange in the SAZ, strengthening our findings based on above extensive proxy reconstructions.

## Discussion

It is widely thought that declined biological pump efficiency, possibly owing to reducing nutrient utilization associated with dust-borne iron deposition, enhanced CO_2_ outgassing in the SAZ and hence atmospheric pCO_2_ rises during deglaciations^1,10,11,24^. During HS1 when atmospheric pCO_2_ raised substantially and dust deposition in Antarctica declined dramatically^45^ (Fig. 3), δpCO_2_^BIO^, together with independently measured δ^15^N_FB_, indicates little net biological pump change at site MD97-2106. Over the same period, δpCO_2_^BIO^ shows little increase in response to the reduced iron fertilization inferred from dust deposition in Antarctica^45^ and other examined sites. At two of the examined sites (ODP 1090 and PS2498-1), where opal and lithogenic fluxes are available^24,46–48^, δpCO_2_^BIO^ shows correlation with neither flux during HS1^24,46,47^ (Fig. 3b, c). In addition to local nutrient utilization efficiency, δpCO_2_^BIO^ and δ^15^N_FB_ at a certain site in the SAZ may also be affected by nutrient supplies modulated by shifts of the Southern Ocean fronts^11,21,39,41,42^. Everything else being equal, poleward movements of the Subtropical and Subantarctic fronts and thus the SAZ during HS1 would reduce nutrient supply, with an effect to raise observed nutrient utilization efficiency, at any given location in the SAZ. During HS1, declining local nutrient utilization efficiency deduced from reduced iron fertilization and poleward front shifts would have opposing effects on SAZ δpCO_2_^BIO^, and their combined effects may result in minimal δpCO_2_^BIO^ changes overall. Thus, despite the lack of any δpCO_2_^BIO^ decline, our reconstructions imply a potential role of iron fertilization in affecting surface-water pCO_2_ through nutrient utilization in the SAZ during HS1.

Instead of a dominant biological contribution, our systematic investigations reveal persistent influences of the solubility pump on surface-water pCO_2_ fluctuations in the SAZ under both modern and past conditions. Modern hydrographical data shows that the solubility pump causes surface-water pCO_2_ to fluctuate by ~40 ppm seasonally (Fig. 1). New and published proxy data demonstrates a solubility effect that can modulate surface-water pCO_2_ by ~20–50 ppm on millennial timescales during the last deglaciation (Figs. 2 and 3). The strong solubility pump influence is likely widespread in the SAZ, as shown by >20 ppm surface-water pCO_2_ increase attributable to solubility pump changes during the early deglaciation in a model simulation^28^ (Fig. 4). Notably, in this model simulation, solubility pump changes in the SAZ lead to strong CO_2_ outgassing and, together with other Southern Ocean processes, contribute to a full-scale atmospheric pCO_2_ increase during HS1 without invoking nutrient utilization efficiency changes related to iron fertilization^28^. Based on our analyses on modern data, proxy reconstructions, and modeling outputs, we suggest that a weakened solubility pump, driven by warming, contributed critically to the rising surface-water pCO_2_ in the SAZ and thereby maintaining this region as a CO_2_ source to the atmosphere during the last deglaciation.

In sum, while our δpCO_2_^BIO^ reconstructions, at face value, indicate little biological pump contributions to deglacial SAZ surface-water pCO_2_ variations at various SAZ sites locally, these reconstructions imply iron-related nutrient utilization effects on the deglacial SAZ surface-water pCO_2_ variabilities when the influence of frontal shift is considered. Nevertheless, such an iron-related biological effect appears to be smaller than previously thought. Therefore, the common view that the iron-regulated SAZ biological pump changes substantially contributed to the deglacial atmospheric pCO_2_ rise^8–11^ may need to be re-evaluated. In comparison to the widely recognized biological pump effect, the potential effect of the solubility pump in the SAZ has been previously overlooked when explaining the past atmospheric pCO_2_ changes. Our work demonstrates that the solubility pump plays an indispensable role in modulating SAZ surface-water pCO_2_ under both modern and past conditions. We suggest future works on quantifying the effect of SAZ solubility pump on past and possibly future atmospheric pCO_2_ changes, which would have important implications for our mechanistic understanding of the global carbon cycle.

## Methods

### Trace element and boron isotope analyses

About 30 to 40 shells of planktic foraminifera *G. bulloides* from the 300–355 µm size fraction and >5 mg of *G. bulloides* shells from the 250–355 µm size fraction were picked for trace element and δ^11^B analyses, respectively. These samples were cleaned following the “Mg-cleaning” procedure^50–53^. Measurements of B/Ca and Mg/Ca, along with Al/Ca and Mn/Ca for monitoring contaminants, were performed on an iCAP Inductively coupled plasma-mass spectrometry (ICP-MS) at the Australian National University (ANU), following an established method^51^.

Separation of boron from sample matrices and measurement of δ^11^B on a multi-collector-ICP-MS (MC-ICP-MS) generally follows the method of Foster^31^ with some modifications. The cleaned foraminifera shells were dissolved in 0.5 M HNO_3_, and buffered by 2 M NH_4_Ac, instead of NaAc-HAc mixture, to pH of ~5.5. We changed the buffering solution to eliminate potential matrix contamination of Na on the δ^11^B measurement. The buffered solution was gravitationally dripped into micro-columns, loaded with 20 µL ion exchange resin (Amberlite IRA-743, 63–125 µm size fraction), which was precleaned by 0.5 M HNO_3_ and then boron-free deionized water. These micro-columns were tested by processing reference materials (boric acid solutions, NIST SRM 951 and ERM AE-121, without and with addition of CaCO_3_ matrix; standard carbonates, NEP-3B) and generating values consistent with published values. After rinsing the resin eight times using Milli-Q water, boron was eluted by five aliquots of 90 µL 0.5 M HNO_3_. A sixth aliquot was also added and collected to check for complete boron recovery. Total procedure blanks for each batch of samples were monitored, and were between ~20 and 100 pg.

δ^11^B was measured on a MC-ICP-MS (Neptune Plus) at ANU using a standard bracketing method similar to Foster^29^. Following Farmer et al.^54^, we measured boron blanks before every bracketing standard (NIST SRM 951) and sample. We also introduce water aerosol into the spray chamber through a second nebulizer after every standard/sample measurement in addition to the routine rinse, to flush out boron in order to minimize the memory effect of boron. An analytical block is as follows: flush-blank-standard-flush-blank-sample-flush-blank-standard. With the additional water flushing in between, measured blanks for ^11^B can be kept <1.8% (an average for blanks of all the samples, external standards, and bracketing standards during 5 sessions) of the bracketing standard with 30 ppb of boron. Prior to and during analyses of these samples, repeating measurements of standard materials (NIST SRM 951, ERM AE-121, NEP-3B, and NIST RM 8301f) yield results consistent with their published values (Supplementary Table 2). The external reproducibility is estimated by repeating measurements of standard ERM AE-121 at 30-ppb boron concentration along with the samples (2σ = 0.17‰, *n* = 12). The boron concentration of the standard is chosen to match the expected median concentration of samples. Three of the foraminiferal samples were divided into two subsamples and processed separately from the cleaning step, and standard deviations of these replicated samples range from 0.08 to 0.27‰ (Supplementary Table 3).

During the late LGM and HS1, *G. bulloides* δ^11^B we measured at site MD97-2106 agrees with previous measurements by MC-ICP-MS in the Pacific and Atlantic SAZ^23^ and the Subtropical Southwest Pacific^55^, but is on average ~1‰ lower than those from the same site measured on a Negative Thermal Ionization Mass Spectrometry (N-TIMS) by a previous study^56^ (Supplementary Fig. 3). We tentatively attribute such offsets to potential analytical biases between MC-ICP-MS and N-TIMS that, as shown by a previous study, range from 0.5 to 2.7‰ and appear to enlarge for samples with low B/Ca values^54^. Despite that Moy et al.^56^ show different deglacial δ^11^B and thus surface-water pCO_2_ magnitudes, deglacial δpCO_2_^SOL^ and δpCO_2_^BIO^ calculated using their data show similar patterns to those based on our new data (Supplementary Fig. 3).

### Carbonate chemistry system calculation

*G. bulloides* δ^11^B is converted into δ^11^B of seawater borate (δ^11^B_borate_) using the calibration from Raitzsch, et al.^30^: δ^11^B_borate_ = (δ^11^B_*G. bulloides*_ + 3.58 ± 11.77)/(1.09 ± 0.65). To estimate pH, SST and surface seawater salinity (SSS) are required. SST is estimated from *G. bulloides* Mg/Ca using the calibration of Elderfield and Ganssen^35^. SSS is estimated from the global sea-level change following Foster^31^. To calculate seawater pCO_2_, seawater alkalinity is estimated from the modern seawater SSS-alkalinity relation^20^. We then use the CO2sys script^57^ to calculate seawater pCO_2_ and other carbonate chemistry parameters including DIC. The 2.3–97.7% uncertainties of seawater pCO_2_ are propagated by a 10,000-iteration Monte-Carlo method incorporating uncertainties from δ^11^B (2σ = 0.17‰), SST (2σ = 1 °C), and SSS (2σ = 0.5), and alkalinity which is sourced from SSS and the modern SSS-alkalinity relation^20^. Using a different way to estimate alkalinity (Supplementary Fig. 7) does not substantially affect our calculated seawater pCO_2_ and its decompositions. For published δ^11^B records, surface-water pCO_2_ is recalculated using the same method as this study to be consistent with our methodology. Final uncertainties shown in Figs. 2 and 3 also incorporate age uncertainties. For site MD97-2106, age uncertainty (1σ ranging from 0.2 to 0.8 ka) is derived from the Undatable script^58^, and for published records, a uniform age uncertainty (1σ) of 0.5 ka is assigned.

We partition the total in-situ surface-water pCO_2_ changes (δpCO_2_^TOTAL^) into two components: solubility-driven (δpCO_2_^SOL^) and biology-driven (δpCO_2_^BIO^) components. In the main text, δpCO_2_^SOL^ is derived first, and the different between δpCO_2_^TOTAL^ and δpCO_2_^SOL^ is defined as δpCO_2_^BIO^. Here, we provide an alternative method to derive δpCO_2_^BIO^ first and subsequently δpCO_2_^SOL^. Firstly, δpCO_2_^TOTAL^ is calculate the same way as described in the main text. Secondly, we use DIC and alkalinity values (the same as those used for in-situ pCO_2_ calculations), but constant SST and SSS values at 18 ka to calculate new surface-water pCO_2_. It is important to note that DIC and alkalinity are used as intermediate parameters for calculations, and their values do not need to be accurately quantified to yield well-quantified new pCO_2_ values. This is because DIC and alkalinity are inherently linked given constraint from pH (see Yu et al.^59^ for detailed discussions). Thirdly, δpCO_2_^BIO^ is calculated by changes in the newly calculated surface-water pCO_2_ relative to 18 ka. Fourthly, δpCO_2_^SOL^ is defined by differencing δpCO_2_^TOTAL^ and δpCO_2_^BIO^. As can be seen from Supplementary Fig. 8, the two methods yield consistent results, strengthening reliability of our calculation. The small differences between these two methods are due to the non-linear responses of seawater pCO_2_ to temperature and DIC changes.

For δpCO_2_^SOL^, it may be further partitioned into temperature- and salinity-driven components (δpCO_2_^T^ and δpCO_2_^S^, respectively). We first calculate new surface-water pCO_2_ at each age by using constant DIC, alkalinity, and SSS at 18 ka, but varying SST. Then, δpCO_2_^T^ is derived as changes of the newly calculated pCO_2_ relative to 18 ka. Afterward, δpCO_2_^S^ is defined as the difference between δpCO_2_^SOL^ and δpCO_2_^T^_._ As can be seen from Supplementary Fig. 5, salinity changes tend to counter temperature effect on pCO_2_, but δpCO_2_^S^ are limited to within 10 ppm at all four SAZ sites studied.

Regarding model outputs^28^, we first calculate δpCO_2_^TOTAL^ between 15 ka and 19 ka, simply by differencing in-situ surface-water pCO_2_ values at these times (pCO_2_^in-situ,15ka^ and pCO_2_^in-situ,19ka^, respectively). We re-calculate surface-water pCO_2_ at 15 ka (pCO_2_^recalc,15ka^) using DIC and alkalinity at 15 ka but using SST and SSS at 19 ka. Similar to proxy data, δpCO_2_^BIO^ and δpCO_2_^SOL^ are calculated by: δpCO_2_^BIO^ = pCO_2_^recalc,15ka^ − pCO_2_^in-situ,19ka^ and δpCO_2_^SOL^ = pCO_2_^TOTAL^ − δpCO_2_^BIO^.

For modern hydrological data^29^, monthly δpCO_2_^TOTAL^, δpCO_2_^BIO^, and δpCO_2_^SOL^ represent deviations from annual mean values. δpCO_2_^TOTAL^, δpCO_2_^BIO^, and δpCO_2_^SOL^ are calculated similarly to those for model results described above. For example, monthly δpCO_2_^BIO^ is calculated using monthly alkalinity and DIC but annual mean SST and SSS, while monthly δpCO_2_^SOL^ is calculated using annual mean alkalinity and DIC but with monthly SST and SSS (Fig. S2).

### Foraminifera-bound nitrogen isotope analyses

Sample preparation and measurements of δ^15^N follow protocols in Ren et al.^60^. For each sample, >3 mg of *G. bulloides* shells (250–355 µm size fraction) were picked and crushed under a dissecting microscope. Foraminiferal samples were sonicated in an ultrasonic bath for 5 min with 2% polyphosphate solution, treated in bicarbonate-buffered dithionite−citric acid in an 80 °C water bath for 1 h, and added with basic potassium persulfate solution and autoclaved at 121 °C for 1 h. After every cleaning step, samples were rinsed with deionized water. Cleaned samples were dried overnight at 55 °C. Each sample was weighed (~1.5–3.5 mg) into a combusted glass vial and then dissolved in 3 M HCl to release organic N from the calcite shell. Persulfate oxidation reagent (POR, 0.3 g of 3-time-recrystallized basic potassium persulfate and 0.7 g of NaOH dissolved in 100 mL of deionized water) were added to the dissolved samples which were then autoclaved at 121 °C for 1 h to convert organic N to nitrate. The nitrate concentrations of all POR-oxidized samples were measured to determine N contents after autoclaving using the chemiluminescence method^61^. Average N content of the cleaned calcite samples is 3.07 mmol N per gram. Nitrate concentration of POR and its δ^15^N were constrained by two organic standards (US Geological Survey (USGS) 40, δ^15^N = −4.5‰ vs. air; and a laboratory standard, mixture of 6-aminocaproic acid and glycine, δ^15^N = 5.4‰ vs. air) processed along with samples. Nitrate concentration of POR is 0.2 µM, representing 1–3% of the total N in samples.

The denitrifier method was applied to transform dissolved nitrate and nitrite into nitrous oxide (N_2_O) gas using a naturally occurring denitrifying bacterial strain, *Pseudomonas chlororaphis*, which lacks an active form of the enzyme N_2_O reductase. After degassing of the bacteria for 3 h, 1.5 mL of the bacterial concentrate was added with 5 nmol of samples acidified to pH of 3-7. Two nitrate reference materials (International Atomic Energy Agency NO_3_ reference (IAEA-N3), δ^15^N = 4.7‰ vs. air; and USGS 34, δ^15^N = −1.8‰ vs. air) were processed along with samples to monitor the bacterial conversion and were later repeatedly measured between samples to check the stability of the mass spectrometry.

δ^15^N of foraminiferal samples, together with bacterial blanks and organic standards, were determined by gas chromatography and isotope ratio mass spectrometry using a modified SigBench and MAT253 plus^62^. Due to the small sample size and low N content within foraminifera, no duplicates were made for these samples. Our IAEA-N3 and USGS 34 standards yielded standard deviation (1σ) of 0.06 and 0.07‰, respectively. The standard deviation (1σ) of the organic standards analyzed with these samples is 0.15‰, agreeing with the long-term variability of in-house carbonate standards using homogenized coral samples (±0.25‰). As a result, we assume that the analytical error for the δ^15^N_FB_ is 0.25‰ in our new record.

## Supplementary information

Supplementary Information

Peer Review File

## Data Availability

All data generated in this study have been deposited in the Zenodo database under access code: 10.5281/zenodo.6970032. All data generated in this study are also provided in the Supplementary Information.

## Code availability

Codes used to produce Figs. 1 and 4 are available from Y.D. upon request. Mode output data used to produce Fig. 4 is available from 10.4225/41/5af39aae7960f^28^.

## References

[1] Sigman DM, Hain MP, Haug GH (2010). The polar ocean and glacial cycles in atmospheric CO_2_ concentration. Nature.

[2] Sigman DM, Boyle EA (2000). Glacial/interglacial variations in atmospheric carbon dioxide. Nature.

[3] Yu J (2022). Millennial and centennial CO_2_ release from the Southern Ocean during the last deglaciation. Nat. Geosci..

[4] Yu J (2010). Loss of carbon from the deep sea since the Last Glacial Maximum. Science.

[5] Talley, L. D. *Descriptive Physical Oceanography: An Introduction* (Academic Press, 2011).

[6] Rintoul SR (2018). The global influence of localized dynamics in the Southern Ocean. Nature.

[7] Sarmiento J, Gruber N, Brzezinsld M, Dunne J (2004). High-latitude controls of thermocline nutrients and low lattitude biological productivity. Nature.

[8] Brovkin V, Ganopolski A, Archer D, Rahmstorf S (2007). Lowering of glacial atmospheric CO_2_ in response to changes in oceanic circulation and marine biogeochemistry. Paleoceanography.

[9] Hain MP, Sigman DM, Haug GH (2010). Carbon dioxide effects of Antarctic stratification, North Atlantic Intermediate Water formation, and subantarctic nutrient drawdown during the last ice age: diagnosis and synthesis in a geochemical box model. Glob. Biogeochem. Cycles.

[10] Jaccard SL (2013). Two modes of change in southern ocean productivity over the past million years. Science.

[11] Martínez-García A (2014). Iron fertilization of the Subantarctic Ocean during the last ice age. Science.

[12] Takahashi T (2002). Global sea-air CO_2_ flux based on climatological surface ocean pCO_2_, and seasonal biological and temperature effects. Deep Sea Res. Part II.

[13] Takahashi T (2009). Climatological mean and decadal change in surface ocean pCO_2_, and net sea–air CO_2_ flux over the global oceans. Deep Sea Res. Part II.

[14] Volk, T. & Hoffert, M. I. *The Carbon Cycle and Atmospheric CO*_*2*_*: Natural Variations Archean to Present* Vol. 32, 99–110 (the American Geophysical Union, 1985).

[15] Hain, M., Sigman, D. & Haug, G. in *Treatise on Geochemistry* 2nd ed., Vol. 8, 485–517 (Elsevier, 2014).

[16] Takahashi T, olafsson J, Goddard JG, Chipman DW, Sutherland SC (1993). Seasonal variation of CO_2_ and nutrients in the high‐latitude surface oceans: a comaprative study. Glob. Biogeochem. Cycles.

[17] Khatiwala S, Schmittner A, Muglia J (2019). Air-sea disequilibrium enhances ocean carbon storage during glacial periods. Sci. Adv..

[18] Köhler P, Fischer H, Munhoven G, Zeebe RE (2005). Quantitative interpretation of atmospheric carbon records over the last glacial termination. Glob. Biogeochem. Cycles.

[19] Heinze C, Maier-Reimer E, Winn K (1991). Glacial pCO_2_ reduction by the world ocean: experiments with the hamburg carbon cycle model. Paleoceanography.

[20] Sarmiento, J. L. & Gruber, N. *Ocean Biogeochemical Dynamics* (Princeton University Press, 2006).

[21] Wang XT (2017). Deep-sea coral evidence for lower Southern Ocean surface nitrate concentrations during the last ice age. Proc. Natl Acad. Sci. USA.

[22] Martinez-Boti MA (2015). Boron isotope evidence for oceanic carbon dioxide leakage during the last deglaciation. Nature.

[23] Shuttleworth R (2021). Early deglacial CO_2_ release from the Sub-Antarctic Atlantic and Pacific oceans. Earth Planet. Sci. Lett..

[24] Martinez-Garcia A (2011). Southern Ocean dust-climate coupling over the past four million years. Nature.

[25] Pedro JB (2015). The spatial extent and dynamics of the Antarctic Cold Reversal. Nat. Geosci..

[26] Jouzel J (2007). Orbital and millennial Antarctic climate variability over the past 800,000 years. Science.

[27] Benz V, Esper O, Gersonde R, Lamy F, Tiedemann R (2016). Last Glacial Maximum sea surface temperature and sea-ice extent in the Pacific sector of the Southern Ocean. Quat. Sci. Rev..

[28] Menviel L (2018). Southern Hemisphere westerlies as a driver of the early deglacial atmospheric CO_2_ rise. Nat. Commun..

[29] Gregor L, Gruber N (2021). OceanSODA-ETHZ: a global gridded data set of the surface ocean carbonate system for seasonal to decadal studies of ocean acidification. Earth Syst. Sci. Data.

[30] Raitzsch M (2018). Boron isotope-based seasonal paleo-pH reconstruction for the Southeast Atlantic – a multispecies approach using habitat preference of planktonic foraminifera. Earth Planet. Sci. Lett..

[31] Foster GL (2008). Seawater pH, pCO_2_ and [CO_3_^2−^] variations in the Caribbean Sea over the last 130 kyr: A boron isotope and B/Ca study of planktic foraminifera. Earth Planet. Sci. Lett..

[32] Bostock HC, Hayward BW, Neil HL, Sabaa AT, Scott GH (2015). Changes in the position of the Subtropical Front south of New Zealand since the last glacial period. Paleoceanography.

[33] Sikes EL (2009). Southern Ocean seasonal temperature and Subtropical Front movement on the South Tasman Rise in the late Quaternary. Paleoceanography.

[34] Belkin IM, Gordon AL (1996). Southern Ocean fronts from the Greenwich meridian to Tasmania. J. Geophys. Res. Oceans.

[35] Elderfield H, Ganssen G (2000). Past temperature and δ^18^O of surface ocean waters inferred from foraminiferal Mg/Ca ratios. Nature.

[36] Dai Y, Yu J, Rafter PA (2021). Deglacial ventilation changes in the deep Southwest Pacific. Paleoceanogr. Paleoclimatol.

[37] Ren H (2015). Glacial-to-interglacial changes in nitrate supply and consumption in the subarctic North Pacific from microfossil-bound N isotopes at two trophic levels. Paleoceanography.

[38] Ren H, Sigman DM, Thunell RC, Prokopenko MG (2012). Nitrogen isotopic composition of planktonic foraminifera from the modern ocean and recent sediments. Limnol. Oceanogr..

[39] Studer AS (2018). Increased nutrient supply to the Southern Ocean during the Holocene and its implications for the pre-industrial atmospheric CO_2_ rise. Nat. Geosci..

[40] Studer AS (2015). Antarctic Zone nutrient conditions during the last two glacial cycles. Paleoceanography.

[41] Ai XE (2020). Southern Ocean upwelling, Earth’s obliquity, and glacial-interglacial atmospheric CO_2_ change. Science.

[42] Li T (2020). Rapid shifts in circulation and biogeochemistry of the Southern Ocean during deglacial carbon cycle events. Sci. Adv..

[43] Monnin E (2004). Evidence for substantial accumulation rate variability in Antarctica during the Holocene, through synchronization of CO_2_ in the Taylor Dome, Dome C and DML ice cores. Earth Planet. Sci. Lett..

[44] Marcott SA (2014). Centennial-scale changes in the global carbon cycle during the last deglaciation. Nature.

[45] Lambert F (2008). Dust-climate couplings over the past 800,000 years from the EPICA Dome C ice core. Nature.

[46] Sachs JP, Anderson RF (2003). Fidelity of alkenone paleotemperatures in southern Cape Basin sediment drifts. Paleoceanography.

[47] Anderson RF (2014). Biological response to millennial variability of dust and nutrient supply in the Subantarctic South Atlantic Ocean. Philos. Trans. A Math. Phys. Eng. Sci..

[48] Garcia, H. E. et al. World Ocean Atlas 2018. Vol. 4: dissolved inorganic nutrients (phosphate, nitrate and nitrate+nitrite, silicate). A. Mishonov Technical Editor, NOAA Atlas NESDIS 84, 35pp. https://www.ncei.noaa.gov/sites/default/files/2020-04/woa18_vol4.pdf (2019).

[49] Locarnini, R. A. et al. World Ocean Atlas 2018, Volume 1: Temperature. A. Mishonov, Technical Editor. NOAA Atlas NESDIS 81, 52pp. https://www.ncei.noaa.gov/sites/default/files/2020-04/woa18_vol1.pdf (2019).

[50] Barker S, Greaves M, Elderfield H (2003). A study of cleaning procedures used for foraminiferal Mg/Ca paleothermometry. Geochem. Geophys. Geosyst..

[51] Boyle E, Keigwin L (1985). Comparison of Atlantic and Pacific paleochemical records for the last 215,000 years: Changes in deep ocean circulation and chemical inventories. Earth Planet. Sci. Lett..

[52] Yu J, Elderfield H, Greaves M, Day J (2007). Preferential dissolution of benthic foraminiferal calcite during laboratory reductive cleaning. Geochem. Geophys. Geosyst..

[53] Yu J, Day J, Greaves M, Elderfield H (2005). Determination of multiple element/calcium ratios in foraminiferal calcite by quadrupole ICP-MS. Geochem. Geophys. Geosyst..

[54] Farmer JR, Hönisch B, Uchikawa J (2016). Single laboratory comparison of MC-ICP-MS and N-TIMS boron isotope analyses in marine carbonates. Chem. Geol..

[55] Shao J (2019). Atmosphere‐ocean CO_2_ exchange across the last deglaciation from the boron isotope proxy. Paleoceanogr. Paleoclimatol..

[56] Moy AD (2019). Varied contribution of the Southern Ocean to deglacial atmospheric CO2 rise. Nat. Geosci..

[57] Lewis, E., Wallace, D. & Allison, L. J. *Program Developed for CO*_*2*_*System Calculations* (Carbon Dioxide Information Analysis Center, managed by Lockheed Martin Energy Research Corporation for the US Department of Energy Tennessee, 1998).

[58] Lougheed BC, Obrochta SP (2019). A rapid, deterministic age-depth modeling routine for geological sequences with inherent depth uncertainty. Paleoceanogr. Paleoclimatol..

[59] Yu J (2019). More efficient North Atlantic carbon pump during the Last Glacial Maximum. Nat. Commun..

[60] Ren H (2017). Impact of glacial/interglacial sea level change on the ocean nitrogen cycle. Proc. Natl Acad. Sci. USA.

[61] Braman RS, Hendrix SA (1989). Nanogram nitrite and nitrate determination in environmental and biological materials by vanadium (III) reduction with chemiluminescence detection. Anal. Chem..

[62] Weigand MA, Foriel J, Barnett B, Oleynik S, Sigman DM (2016). Updates to instrumentation and protocols for isotopic analysis of nitrate by the denitrifier method. Rapid Commun. Mass Spectrom..

