# Supplementary material for: Deglacial Subantarctic CO2 outgassing driven by a weakened solubility pump
Source: Nat Commun. 2022 Sep 3;13:5193. doi: 10.1038/s41467-022-32895-9 (PMC9440897; doi:10.1038/s41467-022-32895-9)
Supplement: Supplementary file 1 — Supplementary Information [file 41467_2022_32895_MOESM1_ESM.pdf]

Supplementary Information for

Deglacial Subantarctic CO_2 outgassing driven by a weakened solubility pump

Yuhao Dai[1,†,*], Jimin Yu[2,1,*], Haojia Ren[3], Xuan Ji[1]

[1]*Research School of Earth Sciences, The Australian National University, Canberra, ACT, Australia*

[2] *Pilot National Laboratory for Marine Science and Technology (Qingdao), Qingdao, China*

[3]*Department of Geosciences, National Taiwan University, Taipei, Taiwan*

[†]*Current address: Department of Geology, Lund University, Lund, Sweden*

**Corresponding authors: yuhao.dai@geol.lu.se; jiminyuanu@gmail.com*

Contents of this file

Supplementary Figures 1 to 10

Supplementary Tables 1 to 4

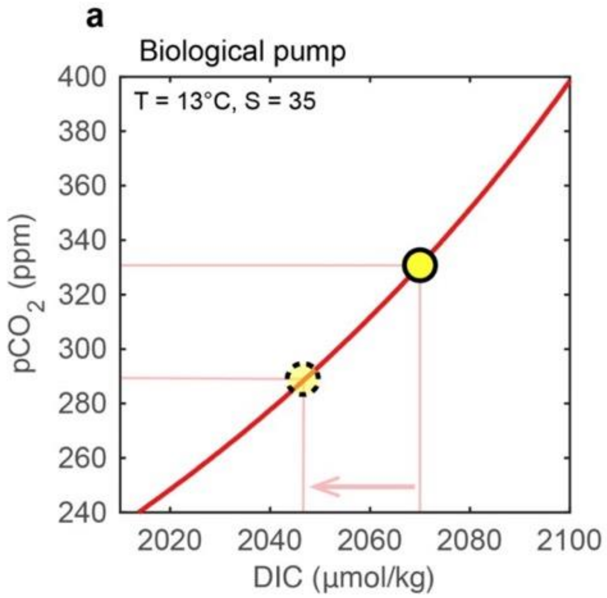

Supplementary Fig. 1 The biological and solubility pumps. a, seawater pCO_2 changes driven by the biological pump at constant temperature and salinity. **b,** seawater pCO_2 changes driven by the solubility pump at constant dissolved inorganic carbon (DIC) and alkalinity. Yellow circles with solid and dashed edges in **a** and **b** illustrate DIC and sea surface temperature (SST) changes required to achieve 40 ppm seawater pCO_2 decrease.

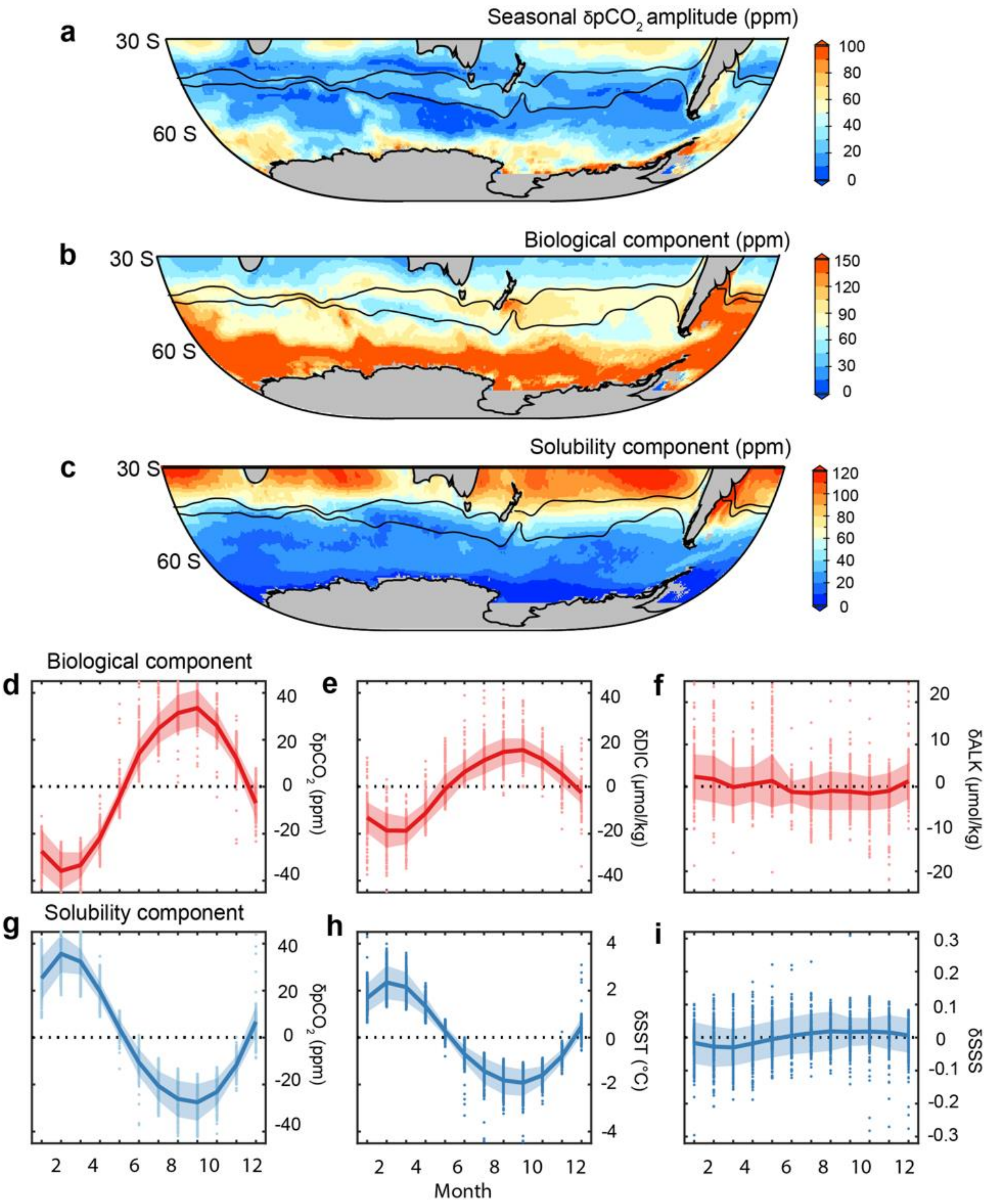

Supplementary Fig. 2 Seasonal variability of the Subantarctic Zone (SAZ) surface-water pCO_2. a, the amplitude of seasonal surface-water pCO_2 (the maximum minus the minimum pCO_2); **b**, the amplitude of seasonal surface-water pCO_2 caused by the biological pump; **c**, the amplitude of seasonal surface-water pCO_2 caused by the solubility pump. **d**, Monthly surface-water pCO_2 variability attributed to biological pump changes within the SAZ, the same as Fig. 1d; **e**, monthly dissolved inorganic carbon (DIC) variability in the SAZ; **f**, monthly alkalinity variability in the SAZ. **g**, Monthly surface-water pCO_2 variability attributed to solubility pump changes within the SAZ, the same as Fig. 1e; **h**, monthly sea surface temperature (SST) variability in the SAZ; **i**, monthly sea surface salinity (SSS) variability in the SAZ. Data is from OceanSODA-ETHZ[1]. In **d-i**, shadings show $\pm 1\sigma$ standard deviation

ranges of observations at discrete locations (represented by dots). Panels **a-c** are made from data presented in Gregor and Gruber: OceanSODA-ETHZ: a global gridded data set of the surface ocean carbonate system for seasonal to decadal studies of ocean acidification, Earth Syst. Sci. Data 13, 777-808 (2021). https://doi.org/10.5194/essd-13-777-2021, https://creativecommons.org/licenses/by/4.0/.

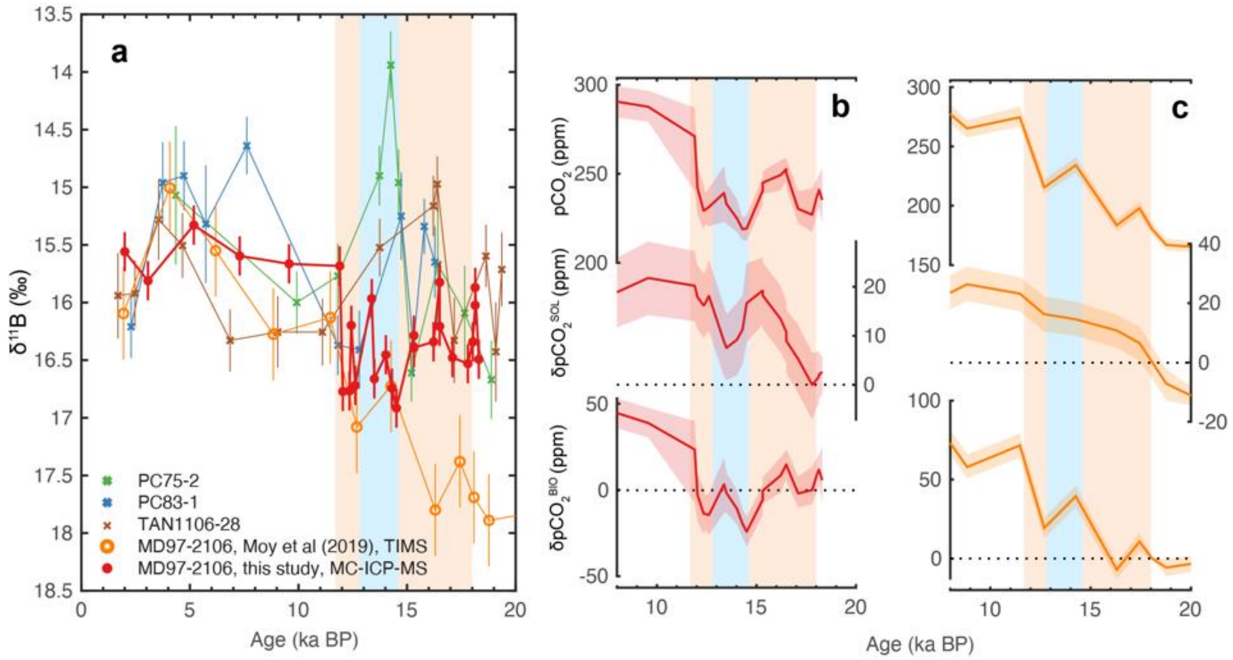

Supplementary Fig. 3 Comparison between surface-water pCO_2 reconstructions between this study and Moy et al[2]. **a,** Comparison of *G. bulloides* $\delta^{11}B$ data with error bars showing $\pm 2\sigma$ uncertainties in the Southwest Pacific during the last deglaciation[2-4]; **b,** surface-water pCO_2, δpCO_2^{SOL}, and δpCO_2^{BIO} based on data from this study; **c,** surface-water pCO_2, δpCO_2^{SOL}, and δpCO_2^{BIO} based on $\delta^{11}B$ measured using Negative Thermal Ionization Mass Spectrometry (N-TIMS) and interpolated sea surface temperature (SST) based on alkenone unsaturation ratios ($U^{K'}_{37}$) from Moy et al.[2]. Data from Moy et al.[2] is adjusted to the age model of this study. Shadings in **b-c** represent 15.9-84.1% (roughly $\pm 1\sigma$) uncertainty ranges of timeseries incorporating uncertainties from measurements, all individual parameters used for calculations, and age models. Despite that Moy et al.[2] show different deglacial $\delta^{11}B$ and thus surface-water pCO_2 magnitudes, deglacial δpCO_2^{SOL} calculated using data from Moy et al.[2] is consistent with conclusions based on our new data.

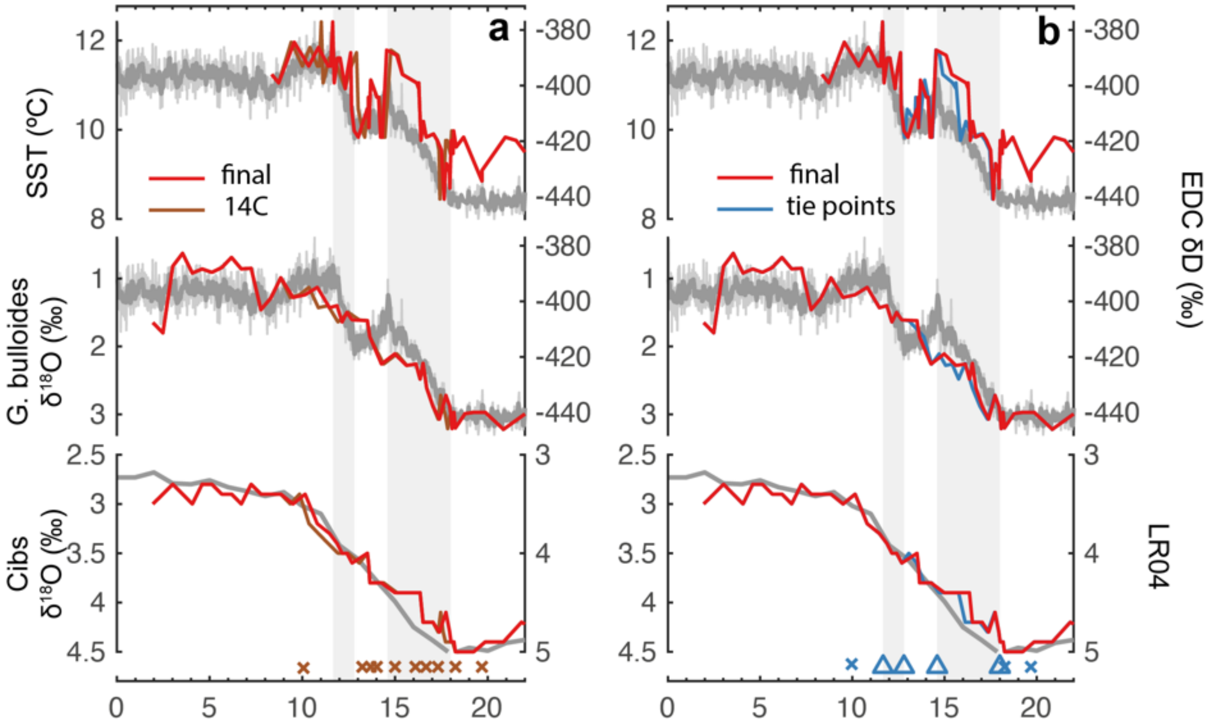

Supplementary Fig. 4 Comparisons of the chosen age model to two alternative age models based on only radiocarbon dates (a) and only SST-δD tie-points (b) during the last deglaciation. The red curves show data in the age model presented in the main text, and brown and blue curves show data in alternative age models. At the bottom of the two panels, crosses and triangles represent tie-points based on radiocarbon and SST-δD tie-points, respectively. To illustrate the consistency between different age models, Mg/Ca-based SST (This study), planktic oxygen isotopes[5], and benthic oxygen isotopes[5] using varying age models are compared to the EDC δD[6] and the global benthic oxygen isotope stack (the LR04 curve[7]).

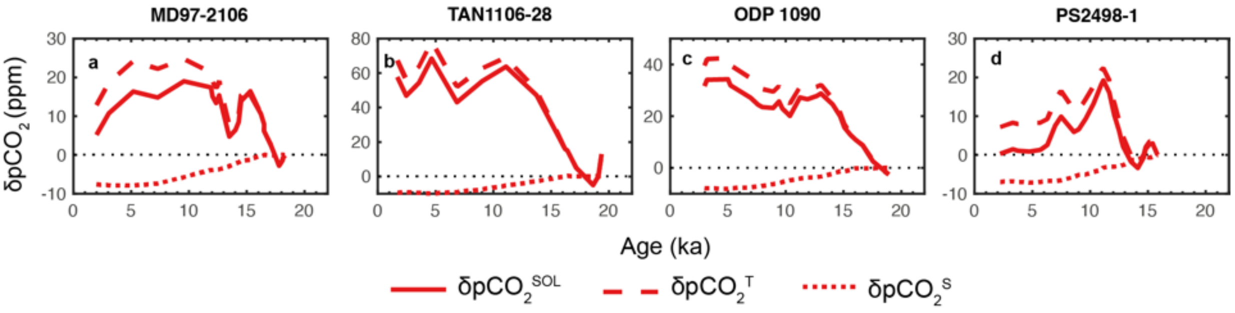

Supplementary Fig. 5 Decomposition of δpCO_2^{SOL} at all studied SAZ sites into components related to temperature and salinity. a, MD97-1026; **b,** TAN1106-28; **c,** ODP 1090; **d,** PS2498-1. The relatively minor contributions of salinity to δpCO_2^{TOTAL} at all these sites indicate that temperature is the dominant contributor to δpCO_2^{SOL}.

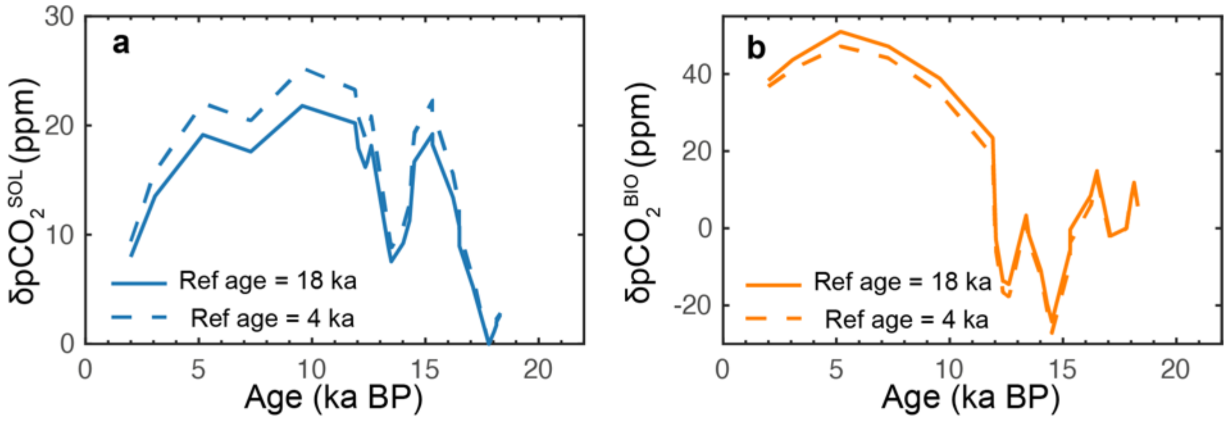

Supplementary Fig. 6 δpCO_2^{TOTAL} decomposition at site MD97-2106 at varying reference ages. a, δpCO_2^{SOL}. **b,** δpCO_2^{BIO}. The solid curves show the calculated values using a reference age of 18 ka that is used in the main text, while the dashed curves show the calculations using a reference age of 4 ka. To enable direct comparison between δpCO_2^{TOTAL} decompositions using different reference ages, pCO_2^{SOL} and pCO_2^{BIO} calculated using a reference age of 4 ka are further converted to the relative change to 18 ka to overlap with calculation using a reference age of 18 ka.

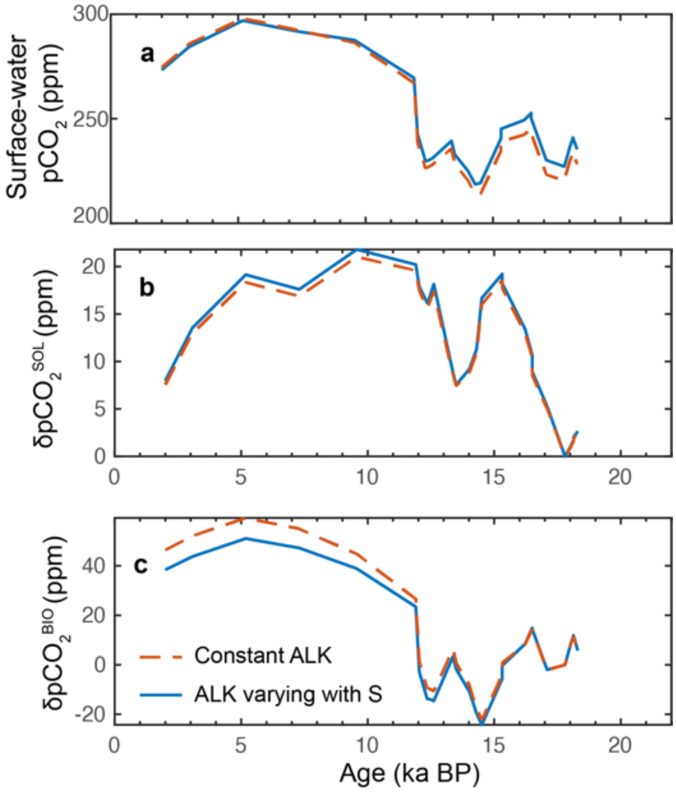

Supplementary Fig. 7 Surface-water pCO_2 calculation and δpCO_2^{TOTAL} decomposition at site MD97-2106 calculated using two difference assumptions about past alkalinity variations. a, surface-water pCO_2; **b**, δpCO_2^{SOL}; **c**, δpCO_2^{BIO}. Blue curves show calculation assuming that the past alkalinity varied linearly with salinity, that is presented in the main text. Red dashed curves show calculation using alkalinity fixed at a constant value (2311 µmol/kg) throughout the deglaciation. As shown here, two methods generate similar results. The general deglacial trends of the calculated surface-water pCO_2 and its solubility and biological components do not rely on the assumed deglacial alkalinity.

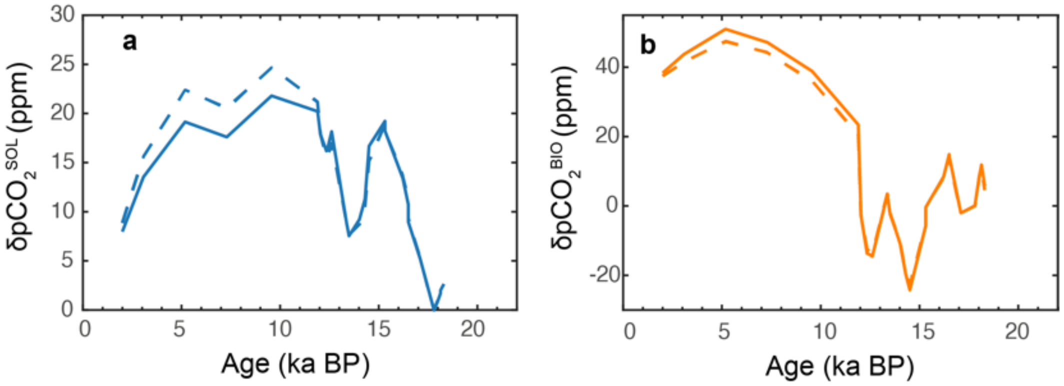

Supplementary Fig. 8 δpCO_2^{TOTAL} decomposition at site MD97-2106 using two methods. a, δpCO_2^{SOL}. **b,** δpCO_2^{BIO}. The solid curves show calculations presented in the main text using a δpCO_2^{TOTAL} partition method deriving δpCO_2^{SOL} directly, while the dashed curves show calculations using an alternative δpCO_2^{TOTAL} partition method deriving δpCO_2^{BIO} directly that is described in the Methods.

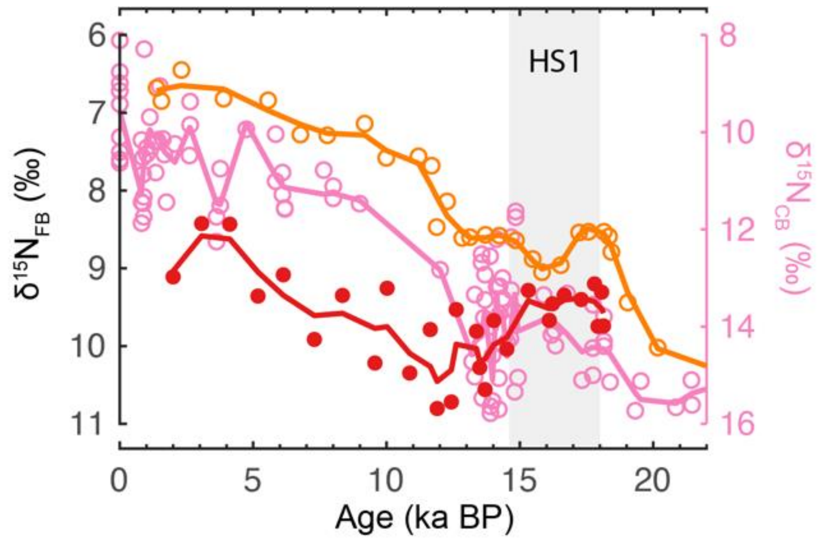

Supplementary Fig. 9 comparison of fossil-bound δ^{15}N records in the SAZ. Red and orange circles indicate foraminifera-bound records from sites MD97-2106 and ODP 1090[8], respectively, and pink circles indicate a coral-bound record from South Tasmania with tighter age controls than sediment cores[9]. During HS1, δ^{15}N changes in all these records are minimal.

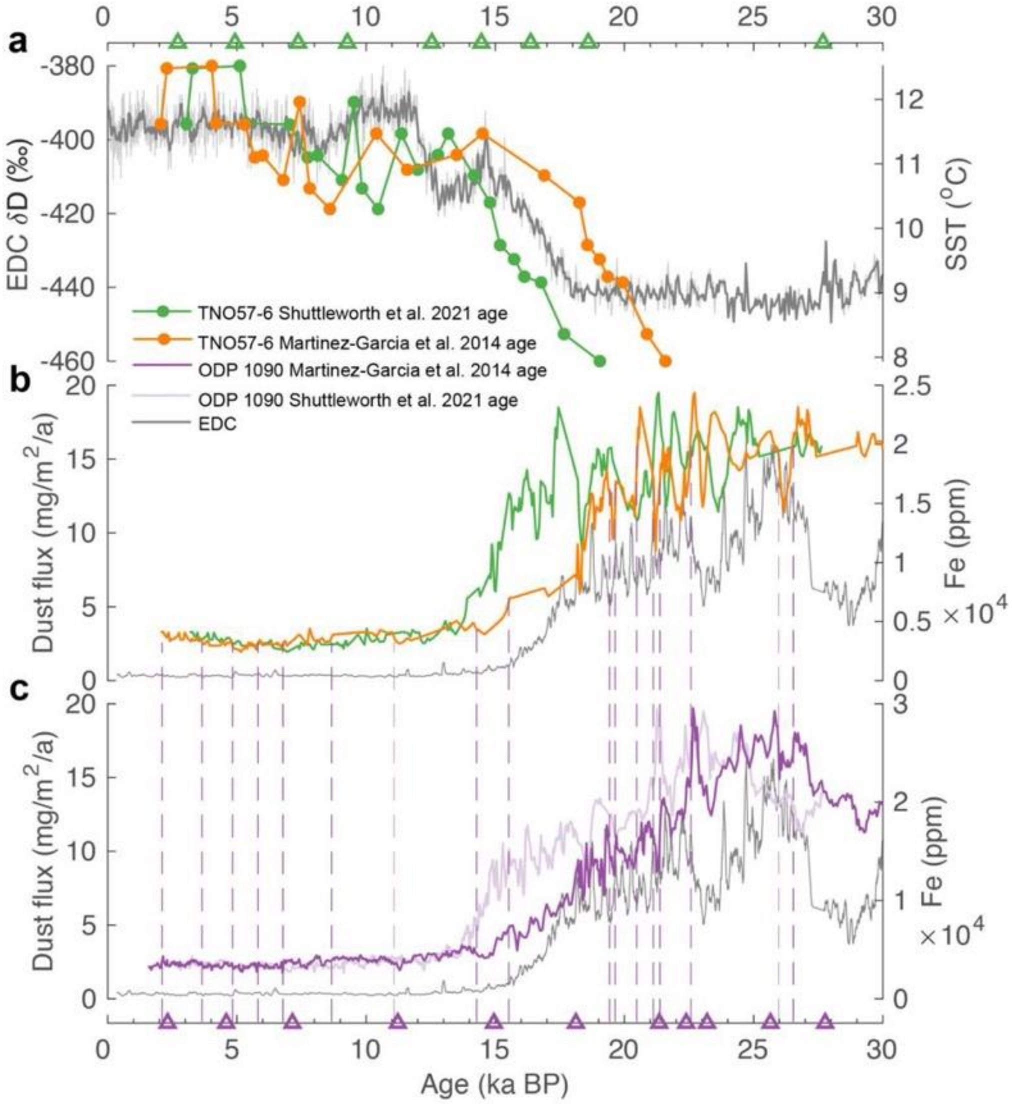

Supplementary Fig. 10 Aligning surface-water pCO_2 reconstruction and $\delta^{15}N_{FB}$ from parallel cores TNO57-6 and ODP 1090. a, Mg/Ca-based SST at TNO57-6 on age models from Ref.[4] (green) and Ref.[8] (orange) compared to EDC δD[6] (grey). **b,** XRF-based Fe concentration at TNO57-6[8] on age models from Ref.[4] (green) and Ref. [8] (orange) compared to EDC dust flux (grey). **c,** XRF-based Fe concentration at ODP 1090[8] on age models from Ref.[4] (light purple) and Ref. [8] (dark purple) compared to EDC dust flux[10] (grey). $\delta^{11}B$-based surface-water pCO_2 reconstruction[4] is from core TNO57-6 and $\delta^{15}N_{FB}$ is from core ODP 1090[8]. While these two cores are often considered the same in the literature because of the proximity and a strong correlation between XRF-based Fe concentrations in these cores, the radiocarbon-based agemodel from Ref. [4] (tie points in **a**) is offset from the age model of Ref. [8] In **b** and **c**, dashed vertical lines are tie points between the two cores based on XRF-based Fe concentrations[8], and the age models of both cores in Ref. [8] were based on the alignment of Fe flux at ODP 1090 to the EDC dust flux[10] (tie points in **c**). In this study, $\delta^{11}B$-based surface-water pCO_2 reconstruction from Ref.[4] is transferred to the age model of Ref. [8], because the age model from Ref. [4] will lead to mismatch between XRF-based Fe concentrations at both cores to the EDC dust flux[10] by up to 5 ka during the LGM, and the last deglaciation (**b, c**).

Supplementary Table 1. Age model of site MD97-2106

Depth top cm	Depth bottom cm	Age yr	Age error yr	Date type	Reservoir age yr	Reservoir age error yr	Reference
3	5	2497	65	^{14}C, *G. inflata*	500	100	Ref. [11]
68	70	8486	45	^{14}C, *G. inflata*	500	100	Ref. [11]
89	92	11670	200	Mg/Ca-δD tie point	N/A	N/A	Ref. [12]
110	112	12800	200	Mg/Ca-δD tie point	N/A	N/A	Ref. [12]
112	113	12525	35	^{14}C, *G. bulloides*	827.5	100	This study
124	125	12615	40	^{14}C, *G. bulloides*	827.5	100	This study
126	127.5	12970	42	^{14}C, *G. bulloides*	827.5	100	This study
130	135	14600	200	Mg/Ca-δD tie point	N/A	N/A	Ref. [12]
134	135	13551	41	^{14}C, *G. bulloides*	827.5	100	This study
144	145	14270	45	^{14}C, *G. bulloides*	827.5	100	This study
158	159.5	14585	40	^{14}C, *G. bulloides*	827.5	100	This study
166.5	168.5	15055	46	^{14}C, *G. bulloides*	827.5	100	This study
172	180	18030	200	Mg/Ca-δD tie point	N/A	N/A	Ref. [12]
203	205	16079	70	^{14}C, *G. inflata*	1155	500	Ref. [11]
217	219	17428	61	^{14}C, *G. bulloides*	1155	500	Ref. [12]
253	255	23530	140	^{14}C, *G. inflata*	1155	500	Ref. [11]

Supplementary Table 2. Measurements of boron isotopes in standard materials.

Standard	δ 11B	[B]	During the run			Long-term lab mean		Published values		
	(‰)	ppb	Mean (‰)	2 s.d. (‰)	n	Mean (‰)	2 s.d. (‰)	Mean (‰)	2 s.d. (‰)	References
ERM AE-121	19.7	29	19.69	0.17	12	19.65	0.19	19.911	0.6	Ref. [13]
	19.8	30								
	19.6	30								
	19.7	30								
	19.7	30								
	19.6	30								
	19.6	30								
	19.7	30								
	19.9	29								
	19.7	29								
	19.7	29								
	19.7	29								
NIST RM 8301f	14.3	29	14.60	0.27	8	14.58	0.19	14.5112	0.17	Ref. [14]
	14.5	29								
	14.7	54								
	14.7	49								
	14.7	51								
	14.7	52								
	14.6	29								
	14.5	29								
NEP-3B			-	-	-	25.67	0.24	25.9613	0.3	Ref. [15]
NIST SRM 951	0.0	30	-0.05	0.05	3					
	0.0	30								
	-0.1	30								

Supplementary Table 3. Record of boron isotopes in *G. bulloides* at site MD97-2106.

Depth top cm	Depth bottom cm	Age ka	Age error 1sd ka	Mg/Ca mmol/mol	SST degree	$\delta^{11}B$ *G.bulloides* ‰	[B] ppb	$\delta^{11}B$ *G.bulloides* 1sd ‰
4	5	2.0	0.2	1.5	10.7	15.6	18	
14	15	3.1	0.4	1.7	11.6	15.8	20	
34	35	5.2	0.8	1.8	12.2	15.3	27	
54	55	7.3	0.5	1.7	11.6	15.6	32	
74	75	9.6	0.3	1.7	12.0	15.7	23	
94	95	11.9	0.2	1.7	11.6	15.7	34	
96	98	12.0	0.2	1.7	11.6	16.8	38	
102	104	12.3	0.2	1.5	10.9	16.8	35	
104	105	12.4	0.2	1.6	11.3	16.2	32	
107	108.5	12.6	0.2	1.7	11.7	16.7	22	
112	114	13.4	0.4	1.5	10.5	16.0	28	
117	119	13.5	0.3	1.5	10.3	16.7	25	
126	127.5	14.0	0.2	1.5	10.7	16.5	20	
129	130	14.3	0.2	1.4	9.8	16.7	34	
131	132.5	14.5	0.2	1.7	11.8	16.9	55	
136	137.5	15.3	0.3	1.6	11.3	16.3	29	
136	137.5					16.4	38	0.07
146	148	16.2	0.3	1.5	10.9	16.3	30	
146	148			1.6				
154	155	16.5	0.2	1.4	10.2	15.8	31	
154	155					16.2	34	0.27
164	165	17.1	0.2	1.4	9.9	16.5	51	
174	175	17.8	0.3	1.3	9.1	16.5	57	
174	175			1.3				
184	185	18.0	0.3	1.4	9.7	16.3	35	
194	195	18.1	0.3	1.3	9.5	16.0	29	
194	195					15.9	21	0.11
204	205	18.3	0.3	1.4	9.6	16.5	59	

Supplementary Table 4. Record of foraminifera bound nitrogen isotopes at site MD97-2106.

top depth cm	bottom depth cm	Age ka	Age error ka	*G.bulloides* $\delta^{15}N$ ‰
4	5	2.0	0.2	9.1
14	15	3.1	0.4	8.4
24	25	4.1	0.6	8.4
34	35	5.2	0.8	9.4
43	44	6.1	0.8	9.1
54	55	7.3	0.5	9.9
64	65	8.3	0.2	9.3
74	75	9.6	0.3	10.2
77	79	10.0	0.4	9.3
84	85	10.9	0.3	10.3
90	91	11.6	0.2	9.8
94	95	11.9	0.2	10.8
104	105	12.4	0.2	10.7
107	108.5	12.6	0.2	9.5
112	114	13.4	0.4	9.8
117	119	13.5	0.3	10.3
124	125	13.7	0.2	10.6
126	127.5	14.0	0.2	9.7
131	132.5	14.5	0.2	10.0
136	137.5	15.3	0.3	9.3
144	145	16.1	0.3	9.7
146	148	16.2	0.3	9.5
158	159	16.7	0.3	9.3
166.5	168	17.3	0.3	9.4
174	175	17.8	0.3	9.2
177	179	17.9	0.3	9.7
187	188.5	18.1	0.3	9.3
194	195	18.1	0.3	9.7

Supplementary References

1 Gregor, L. & Gruber, N. OceanSODA-ETHZ: a global gridded data set of the surface ocean carbonate system for seasonal to decadal studies of ocean acidification. *Earth System Science Data* **13**, 777-808, doi:10.5194/essd-13-777-2021 (2021).

2 Moy, A. D. *et al.* Varied contribution of the Southern Ocean to deglacial atmospheric CO_2 rise. *Nat. Geosci.* **12**, 1006-1011, doi:10.1038/s41561-019-0473-9 (2019).

3 Shao, J. *et al.* Atmosphere-Ocean CO_2 Exchange Across the Last Deglaciation From the Boron Isotope Proxy. *Paleoceanogr Paleoclimatol* **34**, 1650-1670, doi:10.1029/2018pa003498 (2019).

4 Shuttleworth, R. *et al.* Early deglacial CO_2 release from the Sub-Antarctic Atlantic and Pacific oceans. *Earth Planet. Sci. Lett.* **554**, doi:10.1016/j.epsl.2020.116649 (2021).

5 Moy, A., Howard, W. & Gagan, K. Late Quaternary palaeoceanography of the Circumpolar Deep Water from the South Tasman Rise. *J. Quat. Sci.* **21**, 763-777, doi:10.1002/jqs (2006).

6 Jouzel, J. *et al.* Orbital and millennial Antarctic climate variability over the past 800,000 years. *Science* **317**, 793-796 (2007).

7 Lisiecki, L. E. & Raymo, M. E. A Pliocene-Pleistocene stack of 57 globally distributed benthic $\delta^{18}O$ records. *Paleoceanography* **20**, doi:10.1029/2004pa001071 (2005).

8 Martínez-García, A. *et al.* Iron fertilization of the Subantarctic Ocean during the last ice age. *Science* **343**, 1347-1350 (2014).

9 Wang, X. T. *et al.* Deep-sea coral evidence for lower Southern Ocean surface nitrate concentrations during the last ice age. *Proc Natl Acad Sci U S A*, doi:10.1073/pnas.1615718114 (2017).

10 Lambert, F. *et al.* Dust-climate couplings over the past 800,000 years from the EPICA Dome C ice core. *Nature* **452**, 616-619, doi:10.1038/nature06763 (2008).

11 Moy, A., Howard, W. & Gagan, K. Late Quaternary palaeoceanography of the Circumpolar Deep Water from the South Tasman Rise. *J. Quat. Sci.* **21**, 763-777, doi:10.1002/jqs (2006).

12 Dai, Y., Yu, J. & Rafter, P. Deglacial Ventilation Changes in the Deep Southwest Pacific. *Paleoceanogr Paleoclimatol* **36**, doi:10.1029/2020pa004172 (2021).

13 Vogl, J. & Rosner, M. Production and certification of a unique set of isotope and delta reference materials for boron isotope determination in geochemical, environmental and industrial materials. *Geostand. Geoanal. Res.* **36**, 161-175 (2012).

14 Stewart, J. A. *et al.* NIST RM 8301 Boron Isotopes in Marine Carbonate (Simulated Coral and Foraminifera Solutions): Inter-laboratory $\delta^{11}B$ and Trace Element Ratio Value Assignment. *Geostand. Geoanal. Res.* **45**, doi:10.1111/ggr.12363 (2020).

15 McCulloch, M. T., Holcomb, M., Rankenburg, K. & Trotter, J. A. Rapid, high-precision measurements of boron isotopic compositions in marine carbonates. *Rapid Commun Mass Spectrom* **28**, 2704-2712, doi:10.1002/rcm.7065 (2014).
